# Expression of Concern: Alamandine attenuates hepatic fibrosis by regulating autophagy induced by NOX4-dependent ROS

**DOI:** 10.1042/CS-20191235_EOC

**Published:** 2020-07-06

**Keywords:** Alamandine, Angiotensin II, Autophagy, Hepatic fibrosis, NADPH oxidase, Oxidative stress

The Editorial Office has been made aware of potential issues surrounding the scientific validity of this paper, hence has issued an expression of concern to notify readers whilst the Editorial Office investigates.

